# Impact of Caregiver Burden and Care Recipients′ Activities of Daily Living Abilities on Caregivers′ Occupational Dysfunction

**DOI:** 10.1155/oti/1257069

**Published:** 2026-05-12

**Authors:** Keisuke Fujii, Kyosuke Yorozuya, Shumpei Kagayama, Takuya Onaka, Sachiko Miyazaki, Kento Noritake, Yuki Fukumoto

**Affiliations:** ^1^ Faculty of Health Science, Suzuka University of Medical Science, Suzuka, Japan, suzuka-u.ac.jp; ^2^ Faculty of Health Sciences, Bukkyo University, Kyoto, Japan, bukkyo-u.ac.jp; ^3^ Department of Rehabilitation, Geriatric Health Services Facility Yasu Sumireenn, Yasu, Japan; ^4^ Department of Rehabilitation, Geriatric Health Services Facility Asurayasou, Kure, Japan; ^5^ Department of Rehabilitation, Geriatric Health Services Facility Chiryu, Chiryu, Japan; ^6^ Department of Rehabilitation, Tokai Memorial Hospital, Kasugai, Japan; ^7^ Faculty of Health Sciences, Kansai University of Health Sciences, Kumatori, Japan, kansai-u.ac.jp

**Keywords:** caregiving stress, family caregivers, occupational health, older adult care, support interventions

## Abstract

**Introduction:**

Caregiver stress is primarily caused by family and social life disruptions that limit the caregivers′ abilities to engage in meaningful activities, leading to occupational dysfunction. We are aimed at determining the proportion of occupational dysfunction among primary caregivers, its association with caregiver burden, and examining the effects of caregiver burden and care recipients′ activities of daily living (ADL) abilities on primary caregivers′ occupational dysfunction.

**Methods:**

A cross‐sectional, facility‐based survey was used to investigate the proportion of occupational dysfunction among primary caregivers and its association with caregiver burden. A path analysis was conducted to assess the impact of caregiver burden and care recipients′ ADL on occupational dysfunction among primary caregivers. This study included 75 daycare rehabilitation user/primary caregiver pairs. Occupational dysfunction, caregiver burden, and ADL were assessed using the Classification and Assessment of Occupational Dysfunction scale, Zarit burden interview (Japanese version), and the Barthel index, respectively. The impact of caregiver burden and care recipients′ ADL on primary caregivers′ occupational dysfunction was assessed using correlation and path analyses.

**Results:**

The mean age of the primary caregivers and care recipients was 65.2 ± 9.8 years (72.0% were female) and 82.4 ± 7.1 years (48.0% being female), respectively, and 45.3% of the caregivers exhibited occupational dysfunction. Path analysis indicated that a decline in the care recipients′ ADL was associated with an increased caregiver burden and subsequently related to higher occupational dysfunction. The model fit indices were as follows: *χ* ^2^ (df = 1) = 0.010, *p* = 0.091, GFI = 1.000, AGFI = 0.999, CFI = 1.000, and RMSEA = 0.000.

**Conclusions:**

Lower ADL abilities among care recipients were associated with higher caregiver burden and greater occupational dysfunction among primary caregivers. These findings indicate that interventions aimed at reducing caregiver burden and addressing caregiver support needs warrant further investigation.

## 1. Introduction

The global population is aging rapidly, with projections suggesting that the older population will reach approximately 1.015 billion, or 17.8% of the global population, by 2060 [1]. This aging trend is highly pronounced in Japan, with older populations already comprising 29.3% of the population as of October 2024 [2]. Indeed, the number of people in Japan requiring long‐term care continues to increase, reaching 7.06 million by 2024 [3]. Various public services, including rehabilitation, are provided to ensure that older adults with illnesses or disabilities continue to live safely in their communities. However, many care recipients experience a decline in activities of daily living (ADL) [4] and may suffer from dementia [5], making independent living challenging. Despite these obstacles, the support from primary caregivers enables older adults to live in familiar environments. Living in familiar surroundings at home or in the community is said to evoke feelings of attachment to one′s dwelling and community, as well as a sense of security for older adults [6]. Therefore, living in the community is thought to enhance the overall quality of life for these individuals. Indeed, family assistance is often a key factor in allowing patients to return home after hospitalization [7], helping them maintain a sense of autonomy and purpose in daily life. These findings highlight the crucial role of primary caregivers in supporting the community life of older adults.

Simultaneously, the well‐being of primary caregivers is equally important. Caregivers frequently face significant physical and psychological burdens owing to their responsibilities [8, 9]. Disruptions in the caregivers′ family and social life are major contributors to their stress and negatively impact their overall health [10]. From an occupational therapist′s perspective, when caregivers are compelled to adjust their lifestyles and suspend family and social activities due to caregiving, their engagement in meaningful activities becomes limited, potentially resulting in occupational dysfunction. The Model of Human Occupation (MOHO) conceptualizes occupational participation as the result of dynamic interactions among volition, habituation, performance capacity, and environmental factors [11]. Within this framework, sustained caregiving demands can act as environmental and role‐related stressors that disrupt established roles and routines, interfere with occupational identity, and limit engagement in meaningful occupations. Such disruptions, along with imbalances in time use, may reduce occupational participation and contribute to occupational dysfunction. Within this context, caregiver burden can be conceptualized as a proximal subjective appraisal of caregiving demands, whereas occupational dysfunction reflects downstream disruptions in participation and daily functioning. In preventive occupational therapy, occupational dysfunction is defined as a state in which individuals experience negative outcomes due to an inability to adequately perform everyday activities [11]. Occupational dysfunction has been linked to conditions such as depression, burnout [12], loneliness [13], and social isolation [14]. Moreover, chronic caregiver stress has been associated with elevated cortisol levels and other stress‐related biological changes, which are, in turn, linked to an increased risk of cardiovascular disease and mortality [15, 16]. Previous studies have shown that caregivers often neglect their own health due to their caregiving responsibilities, leading to higher rates of chronic illness and reduced life expectancy compared with their noncaregiving peers [17, 18]. Although a substantial amount of research has explored caregiver burden and its impact on daily life, only a few studies have investigated the relationship between caregiver burden and occupational dysfunction.

The ADL abilities of care recipients are another significant factor affecting caregiver burden. A decline in ADL among care recipients may increase the burden on primary caregivers [19, 20], which, in turn, could exacerbate their occupational dysfunction. Thus, when exploring the relationship between caregiver burden and occupational dysfunction among primary caregivers, it is essential to consider the care recipients′ ADL abilities.

In Japan, geriatric health services include community‐based daycare rehabilitation programs that offer multidisciplinary rehabilitation and nursing care to older adults living at home. These facilities play a key role in maintaining functional ability and supporting continued community living, while also shaping the demands placed on family caregivers. Because primary caregivers are actively involved in coordinating and supporting care recipients who use these services, daycare rehabilitation settings provide a relevant and practical context for examining how care recipients′ ADL abilities, caregiver burden, and occupational dysfunction are interrelated.

This study had two primary objectives: first, to clarify the proportion of occupational dysfunction among primary caregivers and examine its association with caregiver burden and second, to use path analysis to explore how caregiver burden and care recipients′ ADL abilities impact occupational dysfunction in primary caregivers. Specifically, this study sought to model and verify the impact of care recipients′ ADL abilities on caregiver burden and caregiver burden on caregivers′ occupational dysfunction. Through this research, we are aimed at providing foundational data that could inform practical interventions to alleviate occupational dysfunction and reduce caregiver burden.

## 2. Methods

### 2.1. Study Design and Ethics

This study used a cross‐sectional survey to clarify the proportion of occupational dysfunction among primary caregivers and to investigate its association with caregiver burden. Additionally, a path analysis was conducted to assess the impact of caregiver burden and care recipients′ ADL on occupational dysfunction among primary caregivers. The study followed the Strengthening the Reporting of Observational Studies in Epidemiology (STROBE) guidelines [21]. The participants were fully informed of the significance and purpose of the study. Written consent was obtained in accordance with the Declaration of Helsinki, and the study was approved by the Kansai University of Health Sciences Research Ethics Review Committee (Ethics Nos. 22–03).

### 2.2. Participants

This study was conducted at three geriatric health service facilities in Japan. Participants included users of daycare rehabilitation services at each facility and their primary caregivers. Data were collected from one facility between August and September 2022 and from the two other facilities between March and April 2023.

**Figure 1:**
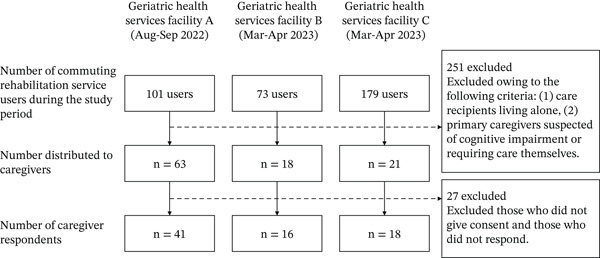
Flowchart of participants.

The inclusion criteria required care recipients to be active users of daycare rehabilitation services. The exclusion criteria were as follows: (1) care recipients living alone, (2) primary caregivers suspected of having cognitive impairment or requiring care, and (3) those who did not provide consent. The participant selection flowchart is shown in Figure 1.

The sample size was determined based on a power analysis with an expected correlation coefficient effect size of 0.4, statistical power of 80%, and significance level of 5%, resulting in a required sample size of 51. Power analysis was conducted using SPSS28.0 (IBM Tokyo, Japan).

### 2.3. Outcome Measures

The primary outcomes for the caregivers were occupational dysfunction and burden. Occupational dysfunction was assessed using the Classification and Assessment of Occupational Dysfunction (CAOD) scale [22]. The CAOD includes 16 items across four domains: occupational imbalance (4 items), occupational deprivation (3 items), occupational alienation (3 items), and occupational marginalization (6 items), with higher scores indicating worse occupational function (range: 16–112). A total CAOD score of 52 or higher was considered indicative of occupational dysfunction [22]. Caregiver burden was measured using the Japanese version of the Zarit burden interview (J‐ZBI) [23], which consists of 22 items, with higher scores indicating greater caregiver burden (range: 0–88). Additional variables for primary caregivers included age, sex, relationship with the care recipient, and frailty, assessed using the Kihon checklist (KCL) [24], a 25‐item questionnaire on daily living, with higher scores indicating greater frailty (range: 0–25).

The main outcome for care recipients was ADL, which was assessed using the Barthel index (BI) [25]. The BI includes 10 items—feeding, transferring, grooming, toilet use, bathing, walking, stair climbing, dressing, bowel control, and bladder control—evaluated by an occupational therapist, with higher scores indicating better ADL abilities (range: 0–100). Additional variables for care recipients included age, sex, and care level, classified within Japan′s Long‐Term Care Insurance system from “Support Level 1” (least severe) to “Care Level 5” (most severe) [26].

### 2.4. Statistical Analysis

**Figure 2:**
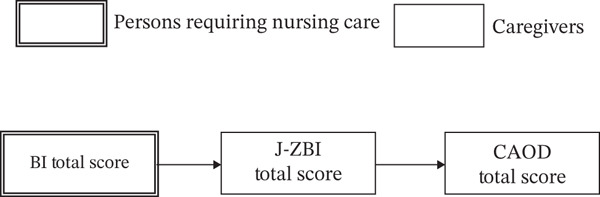
Our hypothetical model.

**Figure 3:**
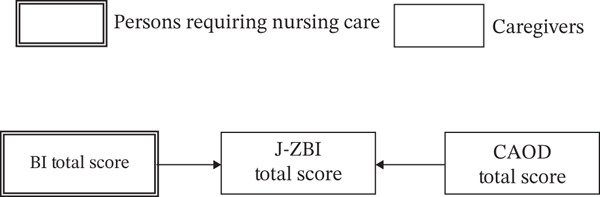
Alternative model to our hypothesis.

Statistical analyses were conducted as follows: (1) Descriptive statistics were calculated for the participants′ characteristics. (2) Correlation analysis was conducted to examine the relationship between the primary caregivers′ caregiver burden and occupational dysfunction. Specifically, the total score and subscale scores of the CAOD, as well as the J‐ZBI score, were calculated for primary caregivers. Furthermore, a correlation matrix was created for the J‐ZBI score, the total CAOD score of primary caregivers, and the BI score of care recipients. (3) Based on the study hypothesis (Figure 2), path analysis was performed to assess the effects of caregiver burden and care recipients′ ADL abilities on occupational dysfunction among primary caregivers. The observed variables and error terms are represented by squares and circles, respectively. (4) Additionally, a reverse causality model hypothesizing that caregiver burden influenced ADL abilities and occupational dysfunction was tested (Figure 3). The model fit indices for all path analyses included the goodness of fit index (GFI), adjusted goodness of fit index (AGFI), comparative fit index (CFI), root mean square error of approximation (RMSEA), and consistent Akaike information criterion (CAIC). A model was considered to have a good fit if GFI > 0.95, AGFI > 0.90, and RMSEA < 0.05, and an acceptable fit if GFI > 0.90, AGFI > 0.85, and RMSEA < 0.08 [27]. The generalized least squares estimation was used for all path analyses. Statistical analysis was conducted using SPSS28.0 (IBM Tokyo) and Amos 19.0 (IBM Tokyo). The statistical significance level was set at 5%.

## 3. Results

The participant selection process is illustrated in Figure 1. In total, 75 pairs of daycare rehabilitation users and their primary caregivers were included as study participants. The mean age of primary caregivers was 65.2 ± 9.8 years, and 72.0% were female. The mean age of care recipients was 82.4 ± 7.1 years, with 48.0% being female. The basic characteristics of primary caregivers and care recipients are presented in Table 1. Among primary caregivers, 45.3% (34 individuals) were identified as having occupational dysfunction.

**Table 1:** Characteristics of the study participants (*N* = 75 pairs).

	People requiring nursing care (*m* *e* *a* *n* ± *S* *D*)	Caregivers (*m* *e* *a* *n* ± *S* *D*)
Age, years	82.9 ± 9.5	69.8 ± 10.5
Female, % (*n*)	45.3 (34)	72.0 (54)
Number of households, number	2.9 ± 1.2	
Classification of certification for long‐term care, % (*n*)		
Support required 1–2	18.7 (14)	
Care Level 1	26.7 (20)	
Care Level 2	25.3 (19)	
Care Level 3	16.0 (12)	
Care Level 4	10.7 (8)	
Care Level 5	2.6 (2)	
BI, score	73.3 ± 22.5	
J‐ZBI, score		27.4 ± 16.9
CAOD, total score, score		48.5 ± 23.1
Occupational imbalance, score		13.8 ± 7.2
Occupational deprivation, score		10.4 ± 5.9
Occupational alienation, score		9.6 ± 5.0
Occupational marginalization, score		14.9 ± 7.6
Occupational dysfunction, presence, % (*n*)		45.3 (34)
KCL, score		6.3 ± 4.0

Abbreviations: BI, Barthel index; CAOD, Classification and Assessment of Occupational Dysfunction Scale; J‐ZBI, Japanese version of the Zarit Caregiver Burden Interview; KCL, Kihon Checklist.

The correlation coefficient (*ρ*) between the total CAOD and J‐ZBI scores was 0.719. Additionally, the correlation coefficients (*ρ*) between caregiver burden and each subscale of occupational dysfunction were as follows: occupational imbalance, 0.670; occupational deprivation, 0.745; occupational alienation, 0.570; and occupational marginalization, 0.643. Furthermore, the correlation matrix for all study variables, including the BI and J‐ZBI total scores, is presented in Table 2.

**Table 2:** Correlation coefficients among the variables included in the analysis.

	BI of persons requiring nursing care		J‐ZBI of caregivers		CAOD of caregivers	
	*ρ*	*p* value	*ρ*	*p* value	*ρ*	*p* value
BI of persons requiring nursing care	1.000		−0.296	0.010	−0.202	0.082
J‐ZBI of caregivers	−0.296	0.010	1.000		0.719	< 0.001
CAOD of caregivers	−0.202	0.082	0.719	< 0.001	1.000	

Abbreviations: BI, Barthel index; CAOD, Classification and Assessment of Occupational Dysfunction Scale; J‐ZBI, Japanese version of the Zarit Caregiver Burden Interview.

**Figure 4:**
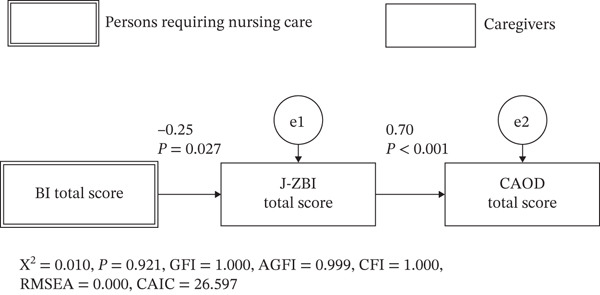
Results of our hypothetical model.

Based on our hypothesis, a path analysis was conducted to examine the effects of caregiver burden and care recipients′ ADL abilities on occupational dysfunction among primary caregivers, as shown in Figure 4. The model fit indices indicated a good fit for the hypothesized model (*χ* ^2^ [df = 1] = 0.010, *p* = 0.091, GFI = 1.000, AGFI = 0.999, CFI = 1.000, RMSEA = 0.000, CAIC = 26.597). The standardized regression coefficient from care recipients′ ADL abilities to caregiver burden was −0.25 (*p* < 0.05), and that from caregiver burden to occupational dysfunction was 0.70 (*p* < 0.05). As shown in Figure 4, the hypothesized model demonstrated that lower ADL abilities among care recipients were significantly associated with higher caregiver burden, which, in turn, was significantly associated with greater occupational dysfunction among primary caregivers. All specified paths in the hypothesized model were statistically significant.

**Figure 5:**
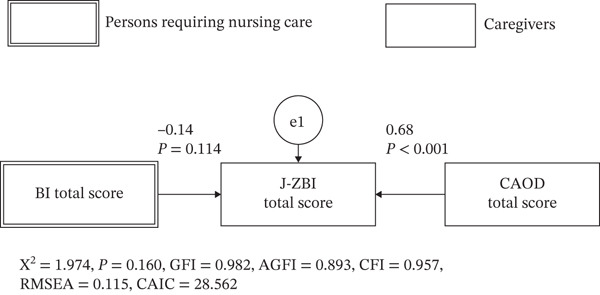
Results of the alternative model to our hypothesis.

Assuming that occupational dysfunction and care recipients′ ADL influence caregiver burden, a reverse causality model—different from the hypothesized model—was tested (Figure 5). The prerequisite for this model, which assumes that the BI of care recipients and the CAOD score of primary caregivers are independently related, was confirmed (*ρ* = −0.202, *p* = 0.082). However, the model fit indices for this alternative model—distinct from the model outlined in our hypothesis—indicated a poor fit (*χ* ^2^ [df = 1] = 2.057, *p* = 0.160, GFI = 0.982, AGFI = 0.893, CFI = 0.957, RMSEA = 0.115, CAIC = 28.562), suggesting that this model did not adequately represent the data. Compared with the hypothesized model, the alternative model, which assumed that occupational dysfunction influences caregiver burden, showed weaker and non‐significant pathways and poorer overall fit indices.

## 4. Discussion

This study examined the effects of caregiver burden and care recipients′ ADL abilities on occupational dysfunction among primary caregivers and revealed two main findings. First, there was a high proportion of occupational dysfunction among primary caregivers, indicating the substantial impact of caregiving on occupational function. Second, a decline in care recipients′ ADL abilities was found to increase caregiver burden, which in turn exacerbated occupational dysfunction. Taken together, these findings suggest a causal structure in which caregiver burden mediates the relationship between care recipients′ ADL abilities and caregivers′ occupational dysfunction. The interrelationships between these factors provide important insights into the development of caregiver support measures.

In this study, 45.3% of the primary caregivers exhibited occupational dysfunction. This finding highlights the impact of caregiver burden on occupational function, especially given the physical and psychological demands, and lifestyle changes imposed by caregiving responsibilities. Furthermore, previous studies have shown that caregivers experience an increase in stress‐related biomarkers. For example, elevated cortisol levels have been reported to be associated with cardiovascular diseases and reduced life expectancy [15–18]. Although it is widely known that caregiver burden negatively impacts health, the findings of this study further clarify its effect on occupational dysfunction. Importantly, occupational dysfunction is better conceptualized as a downstream consequence of sustained caregiver burden, rather than as another stress construct operating in parallel. From a stress‐process perspective, sustained caregiving stress can progressively reshape caregivers′ daily roles and routines, resulting in role loss, role strain, and imbalances in time use. These disruptions in occupational structure may limit engagement in meaningful activities and reduce occupational participation, thereby contributing to occupational dysfunction. Since occupational therapists provide support not only to care recipients but also to their families, it is essential to appropriately assess caregiver burden and consider the possibility that it may lead to occupational dysfunction. The proportion of caregivers with occupational dysfunction (45.3%) was substantially higher than the 15.4% reported in previous studies regarding healthy older adults in Japan, although their caregiving status was unknown [14]. Similarly, the total CAOD score among primary caregivers (48.5 ± 23.1) exceeded that of healthy older adults (30.0 ± 10.5) in prior studies. These findings highlight the significant impact of caregiver burden on occupational function and highlight the urgent need to address caregiver burden as a public health concern. Among the subtypes of occupational dysfunction, occupational deprivation had the strongest association with caregiver burden (*ρ* = 0.719). Occupational deprivation refers to a state in which individuals lack opportunities to engage in meaningful activities due to circumstances beyond their control, such as “having no time to enjoy hobbies,” “lacking opportunities to engage in valued activities,” and “being unable to enjoy things they like” [22]. Disruptions in family and social life are considered primary factors contributing to caregiver stress [10], suggesting that caregivers are often deprived of opportunities to engage in their hobbies and valued activities due to their caregiving responsibilities.

The relationships among care recipients′ ADL abilities, caregiver burden, and occupational dysfunction were further explored. Path analysis revealed that a decline in ADL abilities among care recipients increased caregiver burden and significantly worsened occupational dysfunction among primary caregivers. Specifically, the standardized regression coefficient from ADL abilities decline to caregiver burden was −0.25, whereas that from caregiver burden to occupational dysfunction was 0.70, both statistically significant (*p* < 0.05). This pattern is consistent with stress‐process and caregiving burden frameworks, in which functional dependency acts as a primary stressor and subjective caregiver burden functions as a proximal determinant of caregiver outcomes [28, 29]. Care recipients′ ADL abilities represent caregiving demands placed on caregivers; however, rather than directly affecting occupational dysfunction, these demands exert their influence indirectly, primarily through caregivers′ subjective burden. In contrast, Model 2, which assumed that occupational dysfunction independently influences or parallels caregiver burden, was not supported. This may be explained by the conceptual nature of occupational dysfunction as an accumulated outcome reflecting prolonged disruption of daily occupations, rather than as an antecedent factor shaping burden appraisal. Caregiver burden represents an ongoing subjective evaluation of caregiving demands, whereas occupational dysfunction reflects downstream consequences emerging from sustained burden exposure. Therefore, it is theoretically more plausible that caregiver burden precedes and contributes to occupational dysfunction, rather than the reverse. However, these relationships may be influenced by unmeasured confounding variables. For example, factors such as social support, financial resources, and caregivers′ health status may also affect occupational dysfunction. Moreover, because caregiver‐related variables were assessed using self‐report measures, the observed associations may have been influenced by subjective perceptions. Future studies should employ longitudinal designs or alternative statistical models, such as structural equation modelling with latent variables, to better understand these complex dynamics.

The discussion of clinical implications is presented separately from the interpretation of results. These findings highlight the need for practical interventions to support caregivers.

As the occupational dysfunction of primary caregivers is exacerbated by increased caregiver burden associated with declines in ADL, caregiving services and rehabilitation aimed at improving care recipients′ ADL may positively affect caregivers′ health and occupational abilities. Previous studies have demonstrated that improvements in ADL can reduce caregiver burden by decreasing the level of assistance required [30, 31]. Caregiver burden has been linked to adverse health outcomes, including increased risk of cardiovascular disease [17] and even mortality [18]. Importantly, the present findings suggest that declines in care recipients′ ADL do not directly lead to caregivers′ occupational dysfunction; rather, their impact occurs indirectly through caregivers′ subjective burden. From a clinical perspective, this indicates that interventions solely targeting ADL improvement may be insufficient unless caregivers′ perceived burden is simultaneously addressed. Accordingly, occupational therapy interventions may be particularly effective when they prioritize burden reduction before occupational reconstruction. Many occupational therapists have traditionally focused on improving care recipients′ ADLs [32]. Although this approach benefits clients and their caregivers, the current findings highlight the importance of combining ADL‐focused interventions with strategies explicitly aimed at reducing caregivers′ subjective burden to support the maintenance of caregivers′ occupational and daily life.

However, there may be other methods to alleviate occupational dysfunction beyond ADL abilities improvement alone. Given the association between caregiver burden and occupational dysfunction, additional support at the family, community, and policy levels may be necessary to reduce the caregiver burden. An immediate intervention could be to review the services utilized by care recipients. For example, short‐stay services can provide caregivers with the respite they need. Expanding “respite care”, which offers rest and renewal opportunities for caregivers, and enhancing economic support systems to reduce caregiver burden may also be effective. However, although respite care has been reported to enhance caregiver satisfaction, its effect on stress reduction may be limited [33]. Additionally, its impact has been found to vary depending on the frequency and type of service provided [33]. It has been reported that some caregivers who require respite care do not utilize these services [34]. Recent studies indicate that barriers to service utilization persist, with many caregivers reporting reduced access to or underutilization of formal support services [35]. Despite this, as service utilization has been shown to reduce caregiver burden [36], occupational therapists should consider designing services specifically aimed at alleviating caregiver burden to minimize occupational dysfunction. In addition to system‐level support, occupational therapy interventions can directly address caregivers′ occupational disruption through strategies such as occupational balance training, structured role negotiation, and activity adaptation. Occupational balance training can help caregivers reorganize daily routines and reallocate time for meaningful activities. Role negotiation can assist caregivers in redefining and redistributing responsibilities within the family context. Activity adaptation can support continued engagement in valued occupations despite caregiving demands. Future research should explore how to optimize respite care services to maximize their long‐term effectiveness and identify factors that influence their impact on caregiver burden.

This study clarifies the challenges of caregiver support and provides a foundation for developing support measures that benefit caregivers and recipients. Strengthening interventions and policies to prevent occupational dysfunction among caregivers may be essential to improve the quality of life of caregivers and care recipients.

This study had some limitations. First, although the research was conducted across multiple facilities, it was limited to Japan. Cultural and institutional differences may influence the applicability of these findings in other contexts. Second, as this study employed a cross‐sectional design, causal relationships cannot be established. Future studies should employ longitudinal approaches to validate these findings. Third, participants were recruited exclusively from daycare rehabilitation facilities, which may not represent home‐bound caregivers managing more severe functional impairments. This facility‐based sampling approach may have introduced selection bias and may limit the generalizability of the findings to caregivers in other care contexts. Future studies should include more diverse caregiving settings to improve representativeness. Fourth, primary caregiver outcomes were assessed using self‐report measures, which may be subject to recall bias. Reliance on subjective reporting may have affected the accuracy of the measured levels of caregiver burden and occupational dysfunction. Future studies incorporating objective or multimethod assessments may strengthen measurement validity. Fifth, unmeasured confounding variables, such as caregiver personality traits and access to social resources, may have influenced the results. Future research should incorporate these factors to provide a more comprehensive understanding of caregiver burden and occupational dysfunction.

Moreover, future research should investigate the various factors affecting occupational dysfunction among caregivers and evaluate the effectiveness of specific interventions. In particular, it is essential to explore multifaceted support methods that enhance the quality of life of care recipients, while also protecting the health and well‐being of caregivers.

## 5. Conclusions

This study clarifies the proportion of occupational dysfunction among primary caregivers and its association with caregiver burden. A path analysis was conducted to examine the effects of caregiver burden and care recipients′ ADL on occupational dysfunction among primary caregivers. The results revealed a high proportion of occupational dysfunction among the primary caregivers. Additionally, the findings indicate a decline in ADL among care recipients that is associated with increased caregiver burden, which in turn increases occupational dysfunction. These findings highlight the importance of supporting not only care recipients but also broader client groups, including primary caregivers, when assisting older adults in the community. Rather than demonstrating causality, our findings provide theoretical insight into the associative pathways linking care recipients′ ADL abilities, caregiver burden, and occupational dysfunction. The results underscore the importance of addressing caregiver burden within occupational therapy practice while acknowledging the associative, rather than causal, nature of these relationships and highlight the need for integrative interventions targeting both caregiver burden and occupational participation.

## Author Contributions

**Keisuke Fujii**: conceptualization, methodology, formal analysis, investigation, data curation, and writing—original draft preparation. **Kyosuke Yorozuya**: conceptualization, methodology, investigation, data curation, writing—original draft preparation, and writing—review and editing. **Shumpei Kagayama**: conceptualization, methodology, investigation, and writing—review and editing. **Takuya Onaka**: investigation, data curation, and writing—review and editing. **Sachiko Miyazaki:** investigation, data curation, and writing—review and editing. **Kento Noritake**: formal analysis, investigation, and writing—review and editing. **Yuki Fukumoto**: formal analysis, investigation, and writing—review and editing.

## Funding

This work was supported by the JSPS KAKENHI (Grant Number JP22K17599) and by internal institutional research funds from Kansai University of Health Sciences and Suzuka University of Medical Science.

## Disclosure

The authors take full responsibility for the content of the publication.

## Conflicts of Interest

The authors declare no conflicts of interest.

## Data Availability

The data that support the findings of this study are available from the corresponding author upon reasonable request.
